# Online GESS: prediction of miRNA-like off-target effects in large-scale RNAi screen data by seed region analysis

**DOI:** 10.1186/1471-2105-15-192

**Published:** 2014-06-17

**Authors:** Bahar Yilmazel, Yanhui Hu, Frederic Sigoillot, Jennifer A Smith, Caroline E Shamu, Norbert Perrimon, Stephanie E Mohr

**Affiliations:** 1 Drosophila RNAi Screening Center, Department of Genetics, Harvard Medical School, 77 Avenue Louis Pasteur, Boston, MA 02115, USA; 2 Novartis Institutes for Biomedical Research, Developmental and Molecular Pathways, 250 Massachusetts Avenue, Cambridge, MA 02139, USA; 3 ICCB-Longwood Screening Facility, Harvard Medical School, 250 Longwood Ave, Boston, MA 02115, USA; 4 Howard Hughes Medical Institute, 77 Avenue Louis Pasteur, Boston, MA 02115, USA

**Keywords:** RNAi, Off-target effects, Data analysis, Seed region, miRNA, siRNA, shRNA, High-throughput screening

## Abstract

**Background:**

RNA interference (RNAi) is an effective and important tool used to study gene function. For large-scale screens, RNAi is used to systematically down-regulate genes of interest and analyze their roles in a biological process. However, RNAi is associated with off-target effects (OTEs), including microRNA (miRNA)-like OTEs. The contribution of reagent-specific OTEs to RNAi screen data sets can be significant. In addition, the post-screen validation process is time and labor intensive. Thus, the availability of robust approaches to identify candidate off-targeted transcripts would be beneficial.

**Results:**

Significant efforts have been made to eliminate false positive results attributable to sequence-specific OTEs associated with RNAi. These approaches have included improved algorithms for RNAi reagent design, incorporation of chemical modifications into siRNAs, and the use of various bioinformatics strategies to identify possible OTEs in screen results. Genome-wide Enrichment of Seed Sequence matches (GESS) was developed to identify potential off-targeted transcripts in large-scale screen data by seed-region analysis. Here, we introduce a user-friendly web application that provides researchers a relatively quick and easy way to perform GESS analysis on data from human or mouse cell-based screens using short interfering RNAs (siRNAs) or short hairpin RNAs (shRNAs), as well as for *Drosophila* screens using shRNAs. Online GESS relies on up-to-date transcript sequence annotations for human and mouse genes extracted from NCBI Reference Sequence (RefSeq) and *Drosophila* genes from FlyBase. The tool also accommodates analysis with user-provided reference sequence files.

**Conclusion:**

Online GESS provides a straightforward user interface for genome-wide seed region analysis for human, mouse and *Drosophila* RNAi screen data. With the tool, users can either use a built-in database or provide a database of transcripts for analysis. This makes it possible to analyze RNAi data from any organism for which the user can provide transcript sequences.

## Background

RNA interference (RNAi) is a post-transcriptional gene regulatory mechanism [1] that has been widely used for functional genomics studies both in cell lines and organisms. The synthetic duplexes referred to as small interfering RNAs (siRNAs) or short hairpin RNAs (shRNAs) used for RNAi partner with the RNA Induced Silencing Complex (RISC) to target messenger RNAs for degradation in a sequence-specific manner [2,3]. It has been well established that synthetic duplexes have both on-target activities (reducing expression of intended gene) as well as off-target activities (leading to reduced expression of unintended genes) [4]. A significant fraction of false-positives from RNAi screens is due to off-target effects (OTEs) [5,6]. Many efforts have been made to reduce the number of false positive results due to sequence-specific OTEs, including improved algorithms for RNAi reagent design and incorporation of chemical modifications into siRNAs [7]. Another approach has been to develop bioinformatics strategies to identify possible OTEs in screening results.

OTEs have been linked to the mechanism of action of miRNAs, in which a short sequence of bases 2–8 on the 5′ end of a strand of the RNAi duplex (usually the antisense strand), also called the ‘seed region’, is complementary to the 3′ untranslated regions (UTRs) of multiple mRNAs, causing degradation of their associated transcripts [8,9]. To improve the interpretation of RNAi datasets and to help minimize follow-up experimental efforts, it is important to identify transcripts that are likely to have scored as a result of seed sequence-based targeting. A number of off-target analysis algorithms have been developed and made available to the scientific community. For example, the Haystack algorithm analyzes RNAi off-target effects based on a predictive model trained with published datasets [10]. The model takes into account four types of seed matches and the length of 3’ UTR regions. It requires seed sequence diversity across the dataset and is optimized for large RNAi datasets of sufficient scale with normally distributed scores. Genome-wide Enrichment of Seed Sequence matches (GESS) is another bioinformatics method developed for OTE analysis of RNAi screen datasets [11]. It identifies candidate off-targeted transcripts by investigating the association between matches of the seed regions of RNAi reagents in 3’UTRs with phenotypes observed in large-scale screens. In GESS, RNAi reagents are grouped into two categories: siRNA/shRNAs that score in a screen as “with phenotype” or “active”, and reagents considered “without phenotype” or “inactive”. The algorithm calculates a seed match frequency (SMF) for active and inactive siRNAs/shRNAs for each tested sequence (transcript of a target gene). It is expected that transcripts (and in particular, 3’UTR regions) that are significantly over-represented for seed region matches among active RNAi reagents are more likely to be off-targets. The GESS algorithm has been used successfully to identify off-targeted transcripts in several medium- to large-scale datasets. For example, MAD2 was identified as an off-targeted transcript in a spindle assembly checkpoint components screen and TGFβ-R2 in a screen for novel components of the TGFβ pathway [11]. GESS was also used in analyzing data from a screen for genes required for homologous recombination and predicted RAD51 as a candidate off-targeted gene; RAD51 OTEs were later confirmed experimentally to be responsible for the activity of many siRNAs identified as hits in the primary screen [12].

Previously, MATLAB was used to program and run the GESS algorithm. Standalone versions of the GESS MATLAB code were provided for several operating systems, including Windows, Linux and Mac. Although it is possible to install and use these stand-alone MATLAB versions, it is not easy for biologists lacking programming and informatics expertise to implement GESS in its original form, as the user has to: 1. download and install the program; 2. provide files for the reference sequences; and 3. prepare separate files for siRNAs/shRNAs, phenotype and reagent sequence information. In addition, the run time of the program is not optimal for large files. For example, it can take up to 30 hours to analyze 10,000 siRNAs against 27,500 3’UTRs using the standalone MATLAB version of GESS. Hence, an open, user-friendly online tool with improved performance would be of interest to the scientific community.

## Implementation

Online GESS was developed as a Java web application. Twitter Bootstrap 3 front-end framework and jQuery JavaScript library were used to develop the web pages. At the back end, Online GESS contains reference sequences corresponding to 3’UTRs (the region thought to be the most sensitive to miRNA-off-target effects), 5’UTRs, coding sequences (CDS) or full-length transcripts (including non-coding RNAs) in the human, mouse and *Drosophila* genomes. The human and mouse sequences are obtained from the NCBI RefSeq database. Although these sequences are derived from GenBank records, RefSeq records are non-redundant and have gone through additional levels of validation, annotation, and manual curation. Transcript sequences, as well as CDS and UTR annotations, are retrieved. The *Drosophila* transcript sequences are obtained from FlyBase (flybase.org) [13], a comprehensive database of *Drosophila* information that is curated by experts to ensure quality and includes sequences, gene annotation, mutant alleles and publications. Because curation and annotation of reference sequences is an ongoing effort, we have implemented a mechanism for synchronizing reference sequences with each new RefSeq and FlyBase release [14,15].

After a user uploads their annotated screen results (i.e. sequences of active and, if available, inactive RNAi reagents) in Excel, comma-separated values or tab-delimited text format, the online GESS tool extracts the seed sequences from active and inactive RNAi reagent sequences, then searches the transcript sequences for perfect matches. If a set of inactive RNAi reagents is not provided, the program creates a theoretically inactive set by replacing the first nucleotide of each seed region with the complementary nucleotide. The program then calculates the frequencies of matches among active and inactive RNAi reagents, and identifies transcripts that are significantly enriched among active RNAi reagents using the Fisher exact test and Yates chi-square test. When the sample size is small, the p value from the Fisher’s Exact Test is selected; otherwise, the p value from the Yates Chi Square test is used. Transcripts are then ranked based on the selected p-value. Ranks are later used for calculating multiple hypothesis correction. Three multiple hypothesis correction methods are used in the analysis, the Bonferroni, Bonferroni step-down and Benjamini & Hochberg algorithms, listed in order from most to least stringent correction. Detailed information about the GESS algorithm and analysis methods can be found in the original publication [11].

### User interface

The online GESS application functions as an interface for submitting data and setting parameters for GESS analysis. The output files are sent via e-mail if their size is equal to or smaller than 15 MB. For larger files, a link to download resulting files is provided to user by email. The output files will be available for the user to download for 48 hours.

### User input

In order to perform a GESS analysis, the user has to provide siRNA or shRNA information in one of the required formats (*e.g*. tab or comma separated text file or Excel file). There are two possible layouts for input si/shRNA files. The first requires the sequences of both active and inactive siRNAs/shRNAs, as well as their corresponding phenotype/activity information (see example file at http://www.flyrnai.org/gess/ActiveAndInactiveSiRNAs.txt). The second layout includes only the sequences of active siRNAs/shRNAs, and phenotype/activity information is not needed (all reagents are assumed to be active; see example file at http://www.flyrnai.org/gess/ActivesiRNAs.txt). The user then chooses the correct format for their input file by selecting “Input file contains both active and inactive RNAi reagents” or “Input file contains only active RNAi reagents”. The user also needs to indicate if the input sequences represent the sense (passenger) or anti-sense (guide) strands of the reagents. In addition, the user has to indicate the reagent type, siRNA or shRNA. If shRNA is selected, it is possible for the user to trim the sequences by one to three nucleotides respectively since sequences provided by the source of shRNA library may not reflect the actual mature siRNA strands that are generated by expected canonical dicer cleavage.

The next step is to specify a reference database. As described above, online GESS has built-in reference databases for the human, mouse and *Drosophila* genomes. The user can choose one of the three species and then specify the transcript region(s) to search against. The options are 3’UTR (preferred genomic region for GESS analysis), 5’UTR, CDS, full transcript of protein coding genes, or full transcript region of all genes including non-coding RNA. The user can also choose to upload a custom database file. A custom database file should have FASTA formatted sequences (see example file at http://www.flyrnai.org/gess/customDatabase.txt). For a customized reference database, the program will search for seed matches along the full length of the sequences provided. If the user would like to focus the search to a specific sub-region within a custom reference set, such as 3’UTRs (thought to be the major site of miRNA activity), the user is responsible for uploading only the 3’UTR sequences.

The final step prior to submitting data for processing is to specify any optional parameters. The GESS interface allows users to specify the length of a seed sequence, the minimum number of seed matches to be found in the target sequence, the strand of the RNAi sequence, as well as a statistical threshold value. Currently, the default settings are 7 base pair seed sequence (nucleotides 2–8 from the 5’ end of antisense sequences provided by user), a minimum of one seed match using the anti-sense strand of RNAi only, and a p-value threshold of 0.05 before multiple hypothesis testing correction. The user has the option to perform a control test where each seed sequence of both active and inactive reagents is randomly scrambled. This provides a sense of strength of outliers that may occur at random and more confidence that the significant results are not due to chance. To do this, the user needs to run a parallel test by making corresponding selection under “Advanced Options” at the user interface. This will provide a new set of results and make it possible for users to compare the results obtained for the experimental and control test sets. It is important to note that the program generates only one set of results at a time. Hence, to include a control test in the overall analysis, the control test has to be submitted and run separately.

Online GESS pre-processes the input files and detects mis-formatted records, such as lines missing sequence information, before the analysis starts. If more than 25% of the records are mis-formatted, the error type (see help page at http://www.flyrnai.org/gess/help.jsp) as well as a few examples will be displayed to the user. This feature enables the user to identify errors in their files immediately and fix them. If less than 25% of the records fail pre-processing, the tool continues the analysis, ignoring mis-formatted records in the analysis. The user is then informed via email about the number of RNAi reagents that were ignored in the analysis and their location in the file.

### Output files

A GESS analysis generates two output files. The first file lists the transcripts identified by seed region match to active RNAi reagents and their enrichment scores. By default, this file contains results for all tested transcripts. If the user is not interested in getting the full list, the results of significant transcripts can be obtained by choosing “Only Significant Transcripts” under advanced options. When using a built-in database, each transcript is indicated by its RefSeq accession number, along with a corresponding gene symbol from NCBI or FlyBase. If a custom database is provided, the comment lines from the FASTA file are displayed. This first file also reports the number of active RNAi reagents that have seed matches to a given sequence, the seed match frequency of active reagents, and the p-values according to both Fisher’s Exact and Yates Chi Square tests. The output file also reports the p-value selected for multiple hypothesis correction and the adjusted p-values, as calculated using the Bonferroni, Bonferroni Step-down and Benjamini & Hochberg methods. Finally, the corrected p-value thresholds, as well as statistical significance status of each transcript according to each algorithm, are reported in this file. The second file contains the transcript identifiers and a list of active RNAi reagents that match to them. This file contains only the transcripts with p-values ≤ 0.05. If the analysis fails during input file processing, an email notification is sent to the user (see help page for detailed explanation, http://www.flyrnai.org/gess/help.jsp).

### Run time

The run time of a GESS analysis is dependent upon the input file sizes but in most cases, the analysis is complete within a couple of minutes. For example, in our tests it took two minutes to analyze 10,000 siRNAs against about 68,450 3’UTRs annotated for human genes in RefSeq database (vs61).

### Testing

We compared Online GESS to the standalone MATLAB version using supplementary data from a spindle assembly checkpoint screen as provided in the original GESS publication [11]. The original publication used transcripts from Ensembl as the reference, whereas by default, Online GESS uses transcripts from RefSeq. To do a direct comparison, at Online GESS we uploaded a custom database of 3′UTR sequences from Ensembl as provided in the original publication [11]. We then ran Online GESS using the same parameters as those used in the original publication (a 7mer seed match from either strand) [11] and obtained the same results. Next, we ran another Online GESS analysis with the same parameters using our built-in database of human 3′UTR sequences (by default, this was the current RefSeq release, i.e. v61). The results were the same at the gene level; that is, MAD2 was the only significant outlier. The only differences we observed between results obtained with the standalone MATLAB version and Online GESS were at the transcript level (not at the gene level) and are attributable to differences in the underlying reference data.

## Results and discussion

Using this tool, we analyzed datasets from several publications (Table 1). For the majority of cell lethality screens, Online GESS did not identify any potential off-target genes when the sequences of top hits were analyzed. Cell lethality is a phenotype that can be triggered by a broad range of biological pathways and it is possible that the GESS approach is not sensitive enough to identify potential off-target genes in these cases. On the other hand, for screens measuring phenotypes with more defined molecular mechanisms, such as a spindle assembly checkpoint components screen and a screen for novel components of TGFβ pathway, Online GESS identified potential off-target transcripts (namely, MAD2 in the spindle assembly checkpoint screen and TGFβ-R2 in the TGFβ pathway screen). We also analyzed the ionizing radiation (IR) sensitivity screen published by Hurov et al. [16]. The authors report two datasets for the IR sensitivity phenotype. One is comprised of 850 shRNAs that scored in the primary screen; the other comprises 114 shRNAs that were validated using independent shRNAs. Online GESS did not find any potential OTEs among validated hits but found that ZNF480 and SH3BP2, which appear in the primary hit list, might be off-targeted transcripts. This is consistent with the idea that GESS can help narrow down a list of primary hits and prioritize hits for further validation.

**Table 1:** Datasets tested using the GESS online tool and results

**Study**	**RNAi (species)**	**Phenotype**	**No. active siRNA**	**Statistically significant OT gene**
A genetic screen identifies the Triple T complex required for DNA damage signaling and ATM and ATR stability [16]	shRNA (hs)	Ionizing radiation sensitivity	850	ZNF480 SH3BP2
		Ionizing radiation sensitivity (validated)	114	None
		Ionizing radiation resistance	1080	None
An intermittent live cell imaging screen for siRNA enhancers and suppressors of a kinesin-5 inhibitor [17]	siRNA (hs)	Genes involved in spindle assembly checkpoint (Dharmacon library)	308	MAD2L1
Off-target effects dominate a large-scale RNAi screen for modulators of the TGF-β pathway and reveal microRNA regulation of TGFBR2 [18]	siRNA (hs)	Genes involved in TGF-β signaling (Sloan Kettering Inst. In house library)	409	TGFBR2
Genome-wide siRNA screen identifies SMCX, EP400 and Brd4 as E2-dependent regulators of human papillomavirus oncogene expression [19]	siRNA (hs)	Genes that contribute to the repression of the HPV LCR. (Dharmacon library)	511	None
A genome-wide homologous recombination screen identifies the RNA-binding protein RBMX as a component of the DNA-damage response [12]	siRNA (hs)	Regulator of homologous recombination (Dharmacon library)	510	Rad51
		Regulator of homologous recombination (Ambion library)	187	None
Kinase requirements in human cells: I. Comparing kinase requirements across various cell types [20]	TRC shRNA (hs)	Essential kinases in HeLa and 293 T cell lines.	110	None
A Lentiviral RNAi Library for Human and Mouse Genes Applied to an Array Viral High-Content Screen [21]	TRC shRNA (hs)	Genes required for mitotic progression and proliferation for HT29 cell line	161	None
Highly parallel identification of essential genes in cancer cells [22]	TRC shRNA (hs)	Essential genes for 12 cancer cell lines	182	None
Systematic investigation of genetic vulnerabilities across cancer cell lines reveals lineage-specific dependencies in ovarian cancer [23]	TRC shRNA (hs)	Essential genes specifically for Ovarian cell lines	~1500 (top ~3%)	None
		Essential genes specifically for Colon cell lines	~1500 (top ~3%)	None
		Essential genes specifically for Pancreas cell lines	~1500 (top ~3%)	None
		Essential genes specifically for Esophageal Squamous cell lines	~1500 (top ~3%)	TACO1
Depleting gene activities in early Drosophila embryos with the "maternal-Gal4-shRNA" system [24]	shRNA (dm)	Abnormal embryonic phenotypes	79	None
A regulatory network of Drosophila germline stem cell self-renewal [25]	shRNA (dm)	Abnormal embryonic phenotypes	329	None

Note: The results presented here were obtained using the following parameters: “3’UTR” as genomic region, “antisense/guide” as strand to use, “7” as seed sequence length. The shRNA libraries from The RNAi Consortium (TRC) were trimmed by 2 bp.

In *Drosophila*, short hairpin RNAs have been used for large-scale in vivo screens [26]. We analyzed results from two screens for embryonic phenotypes associated with maternally loaded shRNAs [24,25]. We did not find any potential off-target genes with either dataset, possibly due to the small size of these studies (1000 or 2300 shRNAs, respectively). As more transgenic shRNA screens are done, we anticipate that GESS will prove useful to detect potential off-targeted transcripts that might be associated with *in vivo* Drosophila RNAi screen data.

## Conclusions

RNAi is a powerful tool for systematic study of gene functions but results must be analyzed carefully, as screens are associated with false positive and false negative results. Further validation of results, such as screening with multiple independent RNAi reagents, performing qPCR to verify correlation between knockdown efficiency and phenotypic strength, or RNAi-resistant “rescue” experiments, is time and labor intensive. Detection of potential off-targeted transcripts via automated pre-processing based on our current knowledge of the sources of off-targets, including miRNA-like effects, provides one way to focus limited resources on the most promising candidates. To help support automated detection of off-targeted transcripts in RNAi data, we have implemented a web-based application of seed region analysis for identification of potential off-target transcripts, based on the GESS algorithm. This tool allows users to run off-target analysis with ease. Users can analyze human, mouse or *Drosophila* datasets directly using built-in reference sequence database. In addition, screen data can be analyzed based on a custom reference database, making it possible to analyze RNAi screen results from any organism and at any scale.

## Availability and requirements

• **Project name:** Online GESS

• **Project home page:**http://www.flyrnai.org/gess

• **Operating system(s):** Platform independent

• **Programming language:** Server side: Java, JavaScript

• **Other requirements:** None

• **License:** Not applicable

• **Any restrictions to use by non-academics:** None

## Abbreviations

RNAi: RNA interference; siRNA: Small interfering RNA; shRNA: Short hairpin RNA; miRNA: MicroRNA; OTE: Off-target effect; 5′ UTR: Five prime untranslated region; 3′ UTR: Three prime untranslated region; CDS: Coding Sequences; NCBI: National Center for Biotechnology Information; TRC: The RNAi Consortium.

## Competing interests

The authors declare that they have no competing interests.

## Authors’ contributions

BY designed the UI, implemented the application, analyzed the datasets and drafted the manuscript. YH guided development and testing, analyzed the datasets and drafted the manuscript. FS developed the original algorithm, contributed to tool evaluation and manuscript editing. JAS participated in the UI design, testing and manuscript editing. CES and NP provided guidance and edited the manuscript. SEM provided oversight, guided development and testing, and helped draft the manuscript. All authors read and approved the final manuscript.
